# The Pathology and Treatment of Vertigo

**Published:** 1905-09-30

**Authors:** 


### THE PATHOLOGY AND TREATMENT OF VERTIGO.

In a recent discussion on this subject at the Otological Society of the United Kingdom, Sir Victor Horsley,[1] limiting his remarks to vertigo as a sense of rotation on the part of the patient or to actual rotation as a forced movement, this condition being generally accompanied by nausea, said what was wanted was a knowledge of the site of the initial disturbance. Was this in the periphery—the semicircular canals—and if so was it produced by pressure from the middle ear on the labyrinth or did it arise from disease of the labyrinth itself? Again, the trunk of the vestibular nerve, the cerebellum, the pons, the crus, and finally the cerebral cortex, more particularly that of the temporal lobe, had to be considered as possible situations of the abnormal states responsible for the occurrence of vertigo. He maintained that the condition was strictly analagous to the onset of an epileptic fit or of an attack of trigeminal neuralgia and could only be compared to a condition of things in which there was a summation of stimuli. It was much to be desired that semicircular canal disease could be more accurately localised; this, if possible, would form the basis for more exact central localisation. Passing from the extreme periphery the trunk of the vestibular nerve next demanded consideration, but here disturbance probably never caused vertigo except in the case of new growths. In the medulla the difficulty of exact localisation was extreme. It was almost impossible to differentiate between disease of the trunk of the nerve as this lies under the peduncle of the flocculus and disease of the vestibular nuclei. In every case it was necessary to distinguish two different conditions—namely, true rotation and titubation. Sir Victor suggested that true rotation, that is objective rotation or forced movement, never occurred from mere disease of the middle-ear but only when the vestibular trunk or the wall of the labyrinth was implicated. Considering next the cerebellum, it must be recognised that here they were dealing with two distinct organs, the lateral lobe and the vermis or median portion. The latter was concerned with rotation round a horizontal plane of the body whilst the lateral lobe was related to rotation round the vertical axis of the body, and often in a circle with a large radius. The localisation of a vertigo dependent on one or other of these two organs was thus comparatively precise. Regarding the relation between the cerebellar and cerebral portions of the mechanism of equilibration it was pointed out that the lateral lobes of the cerebellum were not directly united with the cerebrum. They are connected through the middle peduncles with the pons which through the outer part of the crus is associated with the temporal lobe. Lesions of individual parts of the cerebellum and associated tracts and the several effects of these were illustrated by the movements of rabbits shot whilst running away from a gun. In reference to the representation of orientation in the cerebrum attention had hitherto been chiefly paid to the excito-motor or kinæsthetic part of the pallium, the ascending frontal gyrus, but since the most direct anatomical connection between the pons and the cortex was with the posterior two-thirds of the temporal lobe it is here the centre of orientation should be located.

In an attempt at exact anatomical diagnosis in a case of vertigo the first step is to localise the direction of the sense of vertigo and the direction of the forced movement. Next, functional cases must be excluded. Following this is a claim for a complete examination of the nervous system, for by such means only can the localisation of the cause of the disturbance of equilibrium be obtained. Speaking of treatment, Sir Victor said that in middle-ear disease the position simply demanded the application of surgical measures to the diseased bone and mucous membrane. In Menière's disease, that is where in conditions of gout or allied states congestion of the semicircular canals had been diagnosed, he had usually suggested (1) the application of the Weir Mitchell method, (2) hydrobromic acid, and (3) antigouty treatment. He had never had occasion to divide the eighth nerve, though this had been done by other surgeons, and he was quite prepared to do so should the occasion arise. But in his experience one or other of the above lines of treatment had appeared to be sufficient.

In the same discussion Dr. Risien Russell considered vertigo from the medical point of view and in the strict sense of a subjective sensation of movement of the body in some direction in space, whether accompanied or not by actual movement, or a sensation that surrounding objects, though actually stationary, were in movement. In intracranial suppuration due to ear disease it was necessary to recognise that as vertigo might be due to the original ear affection its occurrence might not receive due weight. Dr. Russell, when dealing with vertigo due to organic disease within the skull referred to gross lesions affecting the cerebellum, the temporal lobes, and to extra-medullary lesions at the base of the brain. Vertigo does not distinguish between a lesion at the base of the brain and disease of the ear, for its characters might be the same in both. In a lesion of the left temporo-sphenoidal lobe any indications of word-deafness were of high localising value; careful examination should also be made for any signs of hemiplegia. In gross disease of the cerebellum the vertigo alone might be sufficient to distinguish such a lesion from mere disease of the ear. Experiments showed that in lesions on the same side the direction in which the animal rotated when the eighth nerve was irritated was the opposite to that in which it rotated when a unilateral ablation of the cerebellum had been performed. There were in addition to the above other diseases of the nervous system in which vertigo is sometimes a symptom. In disseminated sclerosis it is a common complaint. In tabes it is much less frequent and is apt to be associated with deafness, from atrophy of the auditory nerve, and thus to come under the notice of the otologist. In idiopathic epilepsy loss of consciousness was of great importance in the diagnosis but here it was essential to recognise that an attack of auditory vertigo could be so sudden as to stun the patient and might cause also loss of consciousness or, at least, so much mental confusion as to make it impossible to distinguish the condition from attacks of *petit mal* associated with vertigo. The vertigo of epileptic origin might be either unattended with such loss or the loss might be so slight as to escape recognition. The relationship of tinnitus and deafness to vertigo becomes of great importance in these cases. Either or both these symptoms might accompany an epileptic attack, but, unless from accidental complications, neither would be present between the attacks. In auditory vertigo, on the other hand, the tendency was for tinnitus and deafness to persist between the attacks. Other causes of vertigo were such functional conditions as hysteria and neurasthenia. In the latter the complaint was rather of giddiness than true vertigo; deafness is exceptional, though tinnitus is not uncommon. Vertigo in hysteria may have all the characters of that due to ear disease. The absence of such disease to examination and the general investigation of the case must be relied on for the correct diagnosis.

[1] Brit. Med. Jour., Sept. 16, 1905.

